# Efficacy of topical administration of prallethrin-permethrin-piperonyl butoxide (Bronco® Equine Fly Spray) for the treatment and control of flies and other nuisance insects of horses

**DOI:** 10.1007/s00436-023-08004-0

**Published:** 2023-11-03

**Authors:** Marco Genchi, Laura Kramer, Gaia Valentini, Giulia Allievi, Lavinia Ciuca, Alice Vismarra

**Affiliations:** 1 Dipartimento Di Scienze Medico-Veterinarie, Parassitologia E Malattie Parassitarie, University of Parma, Via Del Taglio 10, Parma, Italy; 2 Maneggio Le Chianine Dei Tognoli, Via Castel Dell’Aquila, Gragnola, MS Italy; 3 Department of Veterinary Medicine and Animal Productions, University of Naples Federico II, Via Della Veterinaria, 1, Naples, Italy

**Keywords:** Horses, Fly control, Nuisance insect control, Prallethrin, Permethrin

## Abstract

Numerous biting and nuisance insects are a noted cause of discomfort and stress to horses. Pyrethrins and pyrethroids have been used for many years in numerous formulations for the control of insect pests in animals, humans and environment. There are, however, few studies reporting their field efficacy in horses. The aim of the present study was to evaluate the repellent activity of a spray formulation based on prallethrin and permethrin synergized with piperonyl butoxide (BRONCO® Equine Fly Spray, Farnam Companies, Inc., USA) against annoying and harmful insects for horses in field conditions. Nine horses of mixed breed were divided into 2 groups (treatment and control). Pre-treatment insect counts were compared to daily counts for 4 days post-treatment (pt). One minute after the administration of the product (day 0), all the horses were negative for the presence of insects. All counts up to the 6-h pt check remained negative for *Hippobosca equina*, tabanid flies and *Simulium* spp., showing 100% efficacy. This remained above 90% throughout the study. For the *H. equina*, the repellent efficacy remained > 99.7% for all 4 days pt, for tabanid flies > 93.3% and for *Simulium* spp. > 97.4%. The efficacy against *Musca* spp. decreased from 82.2% at day 0 to 62.2% at day 3. Treatment was well-tolerated. In conclusion, despite the low number of tested horses, Bronco® has demonstrated high insecticide and repellent efficacy and a good persistence, maintained for up to 4 days post-treatment, against the most common species of insects harmful for horses.

**Supplementary Information:**

The online version contains supplementary material available at 10.1007/s00436-023-08004-0.

## Introduction

Numerous biting and nuisance insects are a noted cause of discomfort and stress to horses. Among these are the nuisance flies *Musca domestica* and *M. autumnalis* and the biting/blood sucking flies *Stomoxys calcitran* and *Haematobia irritans.* (Diptera: Muscidae). Female tabanid flies (Diptera: Tabanidae) feed on blood and cause seriously painful bites. *Gasterophilus* spp. (Diptera: Oestridae) causes gastrointestinal myiasis, which can be fatal (Hall and Wall 1995) and also provoke considerable irritation and violent reactions due to the intense buzzing of adult flies. *Hippobosca equina* (Diptera: Hippoboscidae), the blood sucking ked, causes cutaneous myiasis. Bloodsucking gnats and midges like *Culicoides* spp. and *Simulium* spp. are also important causes of discomfort and stress in horses. Both cause painful stings, and *Culicoides* spp. is responsible for “sweet itch” (an IgE-mediated, seasonal pruritic dermatosis) (Schaffartzik et al. 2012), while *Simulium* is responsible of painful stings, cause of restlessness in animals and vector of African horse sickness virus (AHSV) and equine encephalosis virus (EEV) (Dennis et al. 2019; Meiswinkel et al. 2004).

Several others can act as vectors of disease (Baldacchino et al. 2014). Flies can transmit bacteria like *Escherichia coli* and *Salmonella* (Zurek and Gorham 2010) and nematodes like *Thelazia* spp. (Lyons et al. 1980) and *Habronema microstoma*, *H. muscae* and *Draschia megastoma* that cause lesions known as “summer sores” (Barlaam et al. 2020).

Pyrethrins and pyrethroids, due to their limited toxicity in mammals, rapid knockdown activity and excellent efficacy (Matsuo and Mori 2012), have been used for many years in numerous formulations including spot-on, pour-on, sprays, dusts, dips and shampoos for the control of insect pests in animals, humans and environment. Their effectiveness against arthropods affecting different animal species is well-known (Franc et al. 2012; Kok et al. 1996; Molina et al. 2012; Rothwell et al. 1999; Stanneck et al. 2012; Williams et al. 1981); however, there is a lack of data regarding their use in horses.

The aim of the present study was to evaluate the repellent activity of a spray formulation based on prallethrin and permethrin synergized with piperonyl butoxide (BRONCO® Equine Fly Spray, Farnam Companies, Inc., USA), against annoying and harmful insects for horses in field conditions. Furthermore, the residual capacity of the product was evaluated for the 4 consecutive days after treatment.

## Materials and methods

### Bronco® chemical composition

The repellent product BRONCO® Equine Fly Spray (Farnam Companies, Inc., 1501 East Woodfield Rd., 200W, Schaumburg, IL 60173, USA) is a topical formulation containing a combination of prallethrin (0.033%), permethrin (0.1%) and piperonyl butoxide (0.5%). The spray is reported as being effective for the control of stable flies, gnats, mosquitoes and ticks and is indicated for use in dogs, horses, ponies, foals, horse barns and stables.

### Animals

The study was conducted in a horse stable of medium dimension (12 horses), located in Gragnola, Tuscany region (210 m above sea level), central Italy. The study was performed from the 15th to the 19th of June 2020. The horses were of mixed breed, different sex and coat colour, aged 3–22 years and with a range of 380–650 kg bodyweight. Neither animals nor the facilities were treated with repellent products for 14 and 30 days before the study, respectively.

The horses were first tested to evaluate attractiveness towards flies (*Musca* spp.). Horses had to be infected with a minimum of 15 flies to be included in the study. The presence of *Hippobosca equina*, Tabanidae, *Simulium* spp., *Culicoides* spp., *Gasterophilus* spp. and mosquitos was also evaluated in order to create more uniform groups.

An informed consent was signed by the horse owners for their use during the study.

### Insect enumeration

**Fig. 1:**
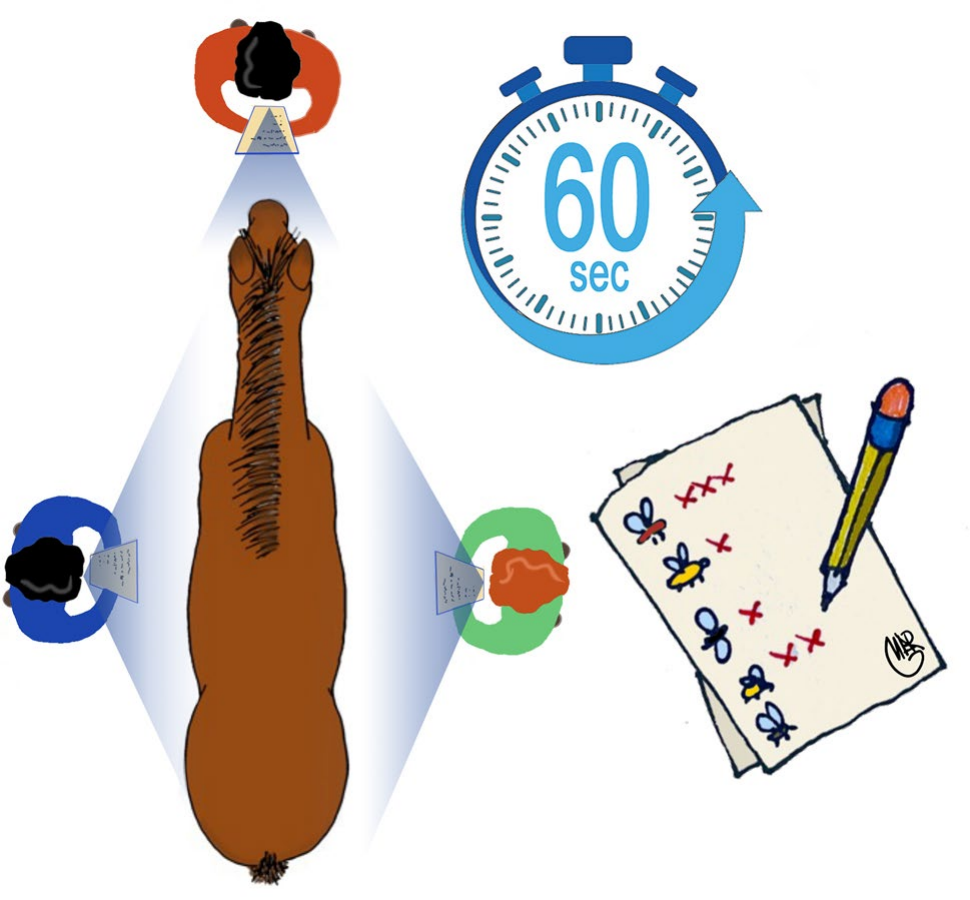
Schematic picture showing the position of the three operators during counting

The presence and number of all the evaluated insects were recorded by three different operators throughout the entire study. One was positioned at the head and the other two at the right and left side of the animals, and they were appointed to count flies and all other insects that remained in contact with horses for at least 5 s. This procedure has been previously reported by several studies and is considered accurate when the same observers are used (Castro et al. 2005; Mullens et al. 2016, 2017). For tabanid flies, those who had completed the blood meal or those who repeatedly tried to do so were counted. The counting time lasted 1 min and was calculated with the help of a stopwatch alarm (Fig. 1). For gnats (*Simulium* spp.), the insects that were inside the ears or nearby were counted, while for *H. equina*, the perineal area and the inner part of the thighs were checked at the end of the counts. All counts were recorded on single personal cards during the 5 days of the study at the different time points (see below). During the counts, some insects were captured and conserved for species identification.

### Treatment protocol

Before treatment (day 1), horses were individually examined to evaluate pre-treatment insect counts. Two enumerations were carried out, in the morning (10:00 am) and in the afternoon (3:00 pm). Based on results, two groups were formed: the treated group (six horses) and the control group (three horses).

Before treatment, horses were carefully brushed to remove dirt and debris from the coat. On day 0, the product to be tested was applied using the spray bottle provided by the manufacturer containing 315 ml of product. All parts of the horse were sprayed, including mane and tail. For the head, the product was administered using a cotton cloth moistened with the product. During treatment, the control group was moved to the opposite side of the structure to avoid any possible contamination.

Insect counts were performed on day 0, before administration of the product (at 10:00 am) and subsequently at 1, 10, 20 and 30 min and at 1, 2, 3, 4, 5 and 6 h after the administration. On days 1, 2 and 3 of the trial, the counts were performed at 10 am, 11 am, 12 pm, 2 pm, 3 pm, 4 pm, 5 pm and 6 pm.

After administration of the product, horses were kept outdoors in proximity of their box, adequately spaced from each other to facilitate insect counting.

Horses were no longer brushed or washed with water after administration of the product and until the end of the trial.

### Statistical analyses

The percentage of effectiveness for each species of insect and for the different time points after treatment was calculated using the average number of insects counted for each horse belonging to the two groups, control and treated, according to the following formula (Abbott 1987):

$$\%\;\mathrm e\mathrm f\mathrm f\mathrm i\mathrm c\mathrm a\mathrm c\mathrm y=\frac{average\;number\;of\;insects\;in\;the\;control\;group-average\;number\;of\;insects\;in\;treated\;group}{average\;number\;of\;insects\;in\;the\;control\;group}\times100$$

### Observations

During the trial, and in particular at each insect count, health status of all animals was evaluated by a veterinarian to assess the presence of any adverse events due to the treatment.

## Results

### Preliminary analyses

On day 1, all selected horses showed ≥ 15 flies required by the protocol to be included in the trial, in particular as regards *Musca* spp. (Suppl. Figures 1–5). Based on these results, the treated group (6 horses: ID 1, 2, 3, 4, 5, 6) and the control group (3 horses: ID 7, 8, 9) were formed.

**Fig. 2:**
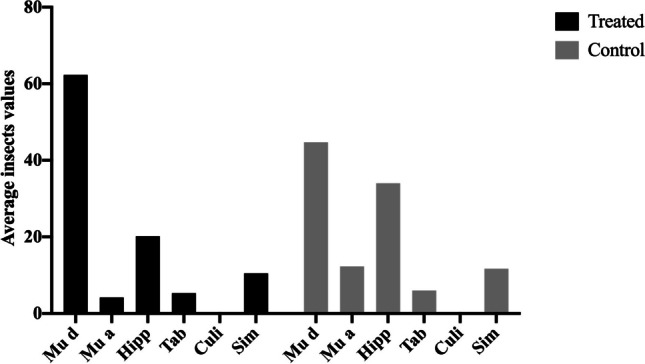
Average value of insects counted at day 1 at different time points in both groups (treated-control)

On day 0, the product was applied to the treatment group. One horse (ID 6, stallion) was extremely nervous and irritable, so it was decided to treat him using a cloth moistened with the product on the entire body, as indicated by the manufacturer. Therefore, the treated group was divided into two further subgroups identified as spray-treated group (five horses: ID 1, 2, 3, 4, 5) and cloth-treated group (1 horse: ID 6) (Fig. 2).

### Control group

The average values of the counts for the control group remained relatively constant for all the insect species during the 4 days of the trial. *M. domestica* maintained an average of 12.6 insects over 4 days, *M. autumnalis* of 8.13, *H. equina* of 15.9, Tabanidae of 2.4 and *Simulium* spp. of 9.3 (Table 1 and Supplementary ST1A).

**Table 1:** Average values for each insect counted during the 4-day trial in the control groups

	ID 7					ID 8					ID 9				
	*Mu d*	*Mu a*	*Hipp*	*Tab*	*Sim*	*Mu d*	*Mu a*	*Hipp*	*Tab*	*Sim*	*Mu d*	*Mu a*	*Hipp*	*Tab*	*Sim*
Day 0	13.9	8.82	19.3	2	8.8	13.3	2.18	18.4	2.5	4.4	16.4	8.09	9.27	2.1	7.9
Day 1	12.8	9.13	15.5	1.3	10	15	4.5	16.5	1.9	5.8	15.4	4.88	10.1	1.4	10
Day 2	12.4	11.9	19	2.3	13	11.8	2.25	17.9	1.8	5.8	12.1	11	12.3	2	12
Day 3	9.5	16	18.1	3.9	14	9.5	4.25	20.5	4	6	9.5	14.6	13.8	4.4	14
Average values for each insects in the control group	*Mu d*	*Mu a*	*Hipp*	*Tab*	*Sim*	Average									
	12.6	8.13	15.9	2.4	9.3	10.35									

Legend: *Mu d*, *Musca domestica*; *Mu a*, *M. autumnalis*; *Hipp*, *Hippobosca equina*; *Tab*, Tabanidae; *Sim*, *Simulium* spp

### Spray-treated group

The average counts for each horse for the spray treatment group at day 0 are reported in Supplementary Table ST1B. One minute after the administration of the product (day 0), all the horses were negative for the presence of insects. All counts up to the 6-h post-treatment (pt) check remained negative for *H. equina*, tabanid flies and *Simulium* spp., showing 100% efficacy. This remained above 90% throughout the study. For the *H. equina*, the repellent efficacy remained > 99.7% for all 4 days pt, for tabanid flies > 93.3% and for *Simulium* spp. > 97.4% (Table 3). Efficacy against *M. domestica* and *M. autumnalis* was 100% after 1-min pt and remained at this level for *M. autumnalis* at 10 and 20 min and 4, 5 and 6 h. The efficacy against *M. domestica* decreased to 89.1% at 10 min pt and only reached 53.5% at 6 h pt. Also in the following days, the repellent efficacy was greater against *M. autumnalis* than against *M. domestica*. In general, the efficacy against *Musca* spp. decreased from 82.2% at day 0 to 62.0% at day 3 (Tables 2, 3 and ST1B).

**Table 2:** Average values for each insects counted during the 4-day trial in the in the treated groups

	ID 1					ID 2					ID 3				
	*Mu d*	*Mu a*	*Hipp*	*Tab*	*Sim*	*Mu d*	*Mu a*	*Hipp*	*Tab*	*Sim*	*Mu d*	*Mu a*	*Hipp*	*Tab*	*Sim*
Day 0	7.6	0.8	0.9	0.1	0.4	2.0	0.2	0.6	0.1	0.3	4.4	0.9	1.4	0.1	0.3
Day 1	8.8	2.5	0.1	0.1	0.4	4.5	0.6	0	0.3	0.4	3.3	1.9	0	0.1	0.3
Day 2	3.8	4.0	0	0.0	0.3	2.0	1.1	0	0.1	0.1	6.6	2.3	0	0.3	0.5
Day 3	5.4	4.5	0	0.6	0.0	2.8	0.4	0	0.3	0.3	8.0	1.5	0.0	0.1	1.3
	ID 4					ID 5					ID 6				
	*Mu d*	*Mu a*	*Hipp*	*Tab*	*Sim*	*Mu d*	*Mu a*	*Hipp*	*Tab*	*Sim*	*Mu d*	*Mu a*	*Hipp*	*Tab*	*Sim*
Day 0	2.8	0.4	3.2	0.4	0.2	4.1	0.1	0.8	0.3	0.5	1.5	0.4	1.6	0.1	0.3
Day 1	3.5	0.9	0.1	0	0	6.3	0.3	0	0.0	0	4.8	0.0	0	0.1	0.1
Day 2	3.9	0.6	0	0	0	5.0	0.5	0	0.3	0	2.6	2.0	0	0.0	0
Day 3	5.3	1.4	0	0	0	8.0	3.0	0	0.3	0	3.6	1.8	0	0.1	0
Average values for each insects in the treated group	*Mu d*	*Mu a*	*Hipp*	*Tab*	*Sim*		Average								
	4.6	1.3	0.4	0.2	0.2		1.34								

Legend: *Mu d*, *Musca domestica*; *Mu a*, *M. autumnalis*; *Hipp*, *Hippobosca equina*; *Tab*, Tabanidae; *Sim*, *Simulium* spp

**Table 3:** Efficacy % of the treatment of *Hippobosca* spp., Tabanidae, *Simulium* spp., *Musca* spp

Day	Average spray-treated group	Cloth-treated group	Average control group	% efficacy spray group	% efficacy cloth group
*Hippobosca* spp.					
0	0	3	105.7	100	97.2
1	0.3	0	112.3	99.7	100
2	0	0	131.0	100	100
3	0	0	139.7	100	100
Tabanidae					
0	0	0	16.7	100	100
1	0.8	1	12.0	93.3	93.3
2	1	0	16.0	93.8	93.8
3	2	1	32.7	93.9	93.9
*Simulium* spp.					
0	0	0	56.7	100	100
1	1.6	1	68.7	97.7	97.7
2	1.4	0	80.7	98.3	98.3
3	2.4	0	92.3	97.4	97.4
*Musca* spp.					
0	27.40	2.00	154.33	82.2	98.7
1	51.80	38.00	164.33	68.5	76.9
2	47.60	37.00	163.67	70.9	77.4
3	64.20	43.00	169.00	62.0	74.6

### Cloth-treated group

Insect counts from horse ID 6 (the cloth-applied product) showed at day 0 two flies after 1, 10 and 20 min pt and three *H. equina* after 1-min pt (Suppl. Table ST1C). Counts then became negative at all further time points until the control at 6 h pt. The average counts at day 0 were 0.6 for *M. domestica* and 0.3 for *H. equina*, while for the other insect species, they were 0 (Suppl. Table ST1C). The efficacy at the different time points on day 0 for *M. autumnalis* shows 100% repellency up to day 1 against *M. domestica*. The efficacy rates during the 4-day pt for *Musca* spp. were 98.7%, 76.9%, 77.4% and 74.6% respectively. For the other insect species, during the 4 days of the test, efficacy was always above 93.3% (Tables 2 and 3).

### General considerations on treated animals

The treatment on day 0 caused very rapid killing activity against *H. equina* in both treated groups. In fact, it was observed that all the specimens that came into contact with the product were no longer able to remain anchored to the skin of the horses, falling down on the ground, showing spastic contractions and then dying. Moreover, in the 8 days following the end of the test, no further *H. equina* specimens were found in any treated horse.

### Insect species identification

The identification of the different species of insects captured during the test highlighted the presence of *M. domestica*, *M. autumnalis*, *H. equina* and *Simulium* spp. For tabanid flies, identified species included *Tabanus sudeticus*, *Philipomyia aprica*, *Haematopota italica*, *Dasyrhamphis anthracinus* and *Chrysops* caecutiens (Baldacchino 2013; Krčmar et al. 2011). *T. sudeticus* and *D. anthracinus* were the most common species. No specimens of *Culicoides* spp., *Gasterophilus* spp. and mosquitoes were identified during the trial.

## Discussion

Pyrethroids are highly toxic to insects and well-tolerated by most mammals. This is due to insects’ increased sodium channel sensitivity, smaller body size and lower body temperature (Bradberry et al. 2005). For this reason, pyrethroids are widely used in different animal species for the control and treatment of fleas, lice, ticks, mosquitoes, flies and other nuisance insects. Their efficacy has been widely reported, and a plethora of products is currently available on the market (Arsenopoulos et al. 2018; Bauer et al. 1992; Beugnet et al. 2016; Franc et al. 2012; Rothwell et al. 1998; Stanneck et al. 2012). There are also many products containing pyrethroids available for the control and treatment of ectoparasites and other nuisance insects in horses (Biggin et al. 1999). However, published reports and field trials on the efficacy of these drugs are lacking. Furthermore, the few articles found on online databases (PubMed, Web of Science using the following strings “Horses AND flies control AND nuisance insects control AND Prallethrin, AND Permethrin”) are almost exclusively about *Culicoides* spp. (Papadopoulos et al. 2010; Schmidtmann et al. 2001; Stevens et al. 1988), with a further two about hematophagous flies (Parashar et al. 1989, 1991).

In the present study, a commercial product containing prallethrin and permethrin synergized with piperonyl butoxide (BRONCO® Equine Fly Spray, Farnam Companies, Inc., USA) was evaluated for its repellent efficacy against a number of nuisance and biting insects in naturally exposed horses. In general, the two treated groups, when compared to the control group, showed a highly significant decrease in the average total number of insects and 1.34 for the two treated groups compared to an average of 10.35 in the control group (Table 2).

The efficacy against Tabanidae and *Simulium* spp. remained high until the end of the study with an efficacy of 93.9% and 97.4%, respectively. This sharp decrease in the number of insects, in particular for *M. domestica*, had an important effect on the treated horses’ behaviour. In fact, in the days after the treatment, the horses appeared much calmer and less stressed. In particular, the continuous movements of the tail, of the head and the activation of furry muscles, commonly used to remove the insects mechanically, was markedly reduced and, in some cases, absent (Suppl. Video 1). Moreover, a notable decrease in tear secretions, often induced by the sharp teeth of *M. autumnalis*, was observed in all treated horses. Furthermore, the sharp decrease in horseflies, sources of painful stings, also contributed positively to the welfare of treated horses.

The data resulting from the administration of the product with the soaked cloth over the whole body of the horse, although very interesting and with very good efficacy for both species of nuisance insects until the end of the test, are difficult to compare considering that only one horse was tested. However, it is interesting to note that the repellent effect for all the species of insects examined was higher, especially for *M. domestica* and *M. autumnalis*, when the product was administered with the cloth. This could be due to the horse’s lesser intrinsic attractiveness to insects and/or a greater penetration of the product into the hair and at the skin level induced by the mechanical action of rubbing during application. Indeed, horse ID 6 showed a much lower mean number of *M. domestica* compared to the other animals in the treated group on day 1 (13.5 compared to a minimum of 28.5 in horse ID 4 and a maximum of 45.5 in horse ID 1).

During the 4 days of the trial, no specimens of *Culicoides* spp., *Gasterophilus* spp. or mosquitoes were observed. For *Culicoides* spp. and mosquitoes, this can be explained by the timing when the counts were done (during the day from 10 am to 6 pm). In fact, these Diptera feed at sunset and during night. For *Gasterophilus* spp., the explanation could be found in the seasonal pattern of the adults of this insect which are present mainly towards the end of summer, while the test took place in June 2020. No clinical signs of local (skin) or general intolerance were observed during all the trial.

Although beyond the scope of the trial, on day 0, several specimens of tick were found prior to treatment (horses ID 2, 3 and 6): 3 adult female of *I. ricinus*, 2 adult females of *Hyalomma* spp. and 8 nymphs. Ticks on day 1 appeared moribund and with a shrivelled cuticle, even though still attached.

## Conclusions

In conclusion, Bronco® has demonstrated high insecticide and repellent efficacy and a good persistence that is maintained for up to 4 days post-treatment against the most common species of insects harmful for horses.

Finally, it is likely that the continued use of Bronco®, both due to its killing effect and to a decrease in the possibility of feeding by bloodsucking insects, could lead to a reduction in the presence of these insects in the environment.

### Supplementary Information

Below is the link to the electronic supplementary material.

Supplementary file1 (JPEG 11192 KB)

Supplementary file2 (JPEG 1361 KB)

Supplementary file3 (JPEG 3197 KB)

Supplementary file4 (JPEG 5899 KB)

Supplementary file5 (JPEG 5313 KB)

Supplementary file6 (DOCX 38 KB)

Supplementary file7 (MOV 44026 KB)

Supplementary file8 (MOV 33725 KB)

## Data Availability

All the data obtained are presented and attached to the manuscript.
