# Supplementary figures and images for: Efficacy of topical administration of prallethrin-permethrin-piperonyl butoxide (Bronco® Equine Fly Spray) for the treatment and control of flies and other nuisance insects of horses
Source: Parasitol Res. 2023 Nov 3;122(12):3139–45. doi: 10.1007/s00436-023-08004-0 (PMC10667147; doi:10.1007/s00436-023-08004-0)

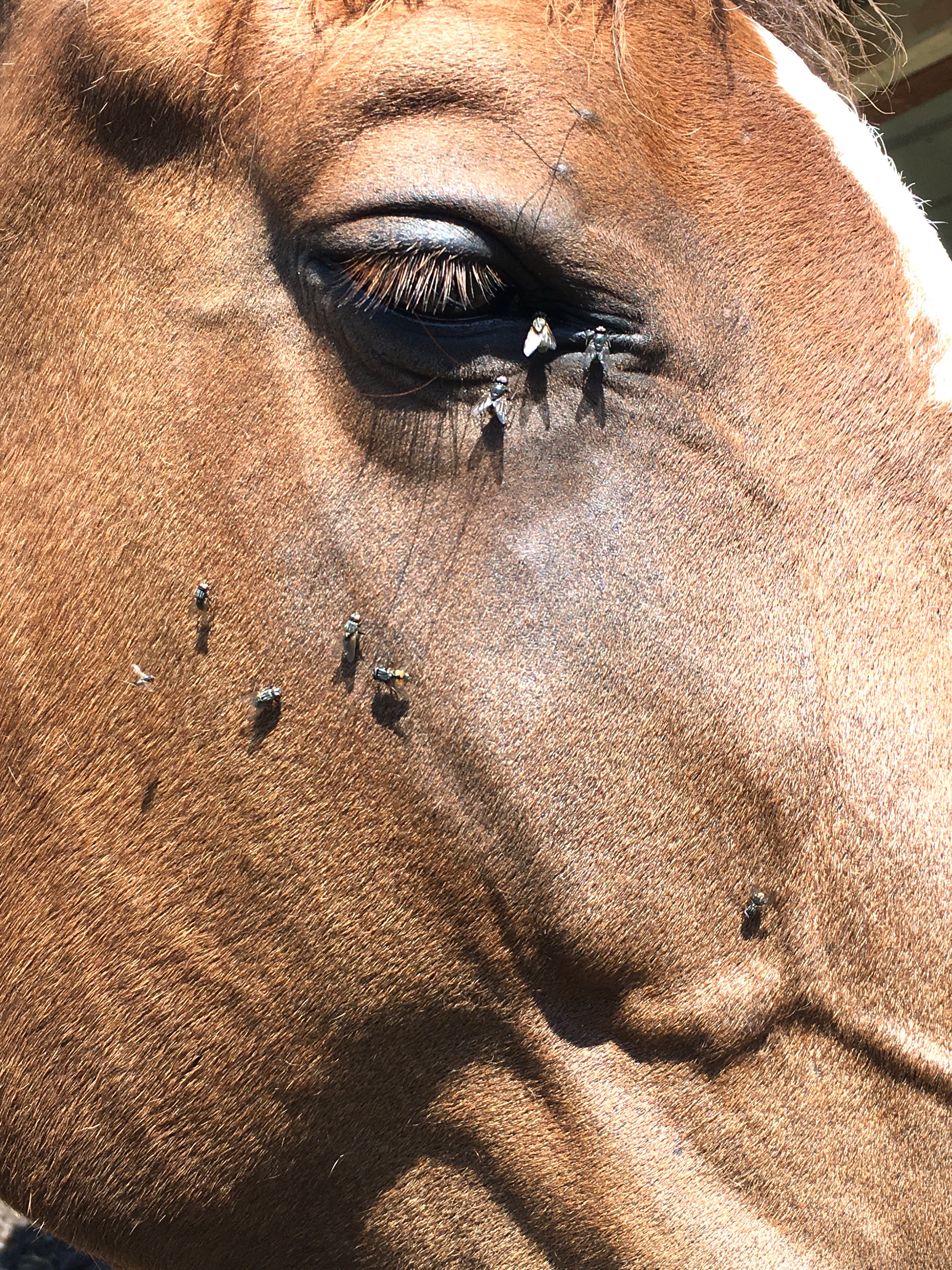

Supplement: Supplementary file 1 — Supplementary file1 (JPEG 11192 KB) [file 436_2023_8004_MOESM1_ESM.jpeg]

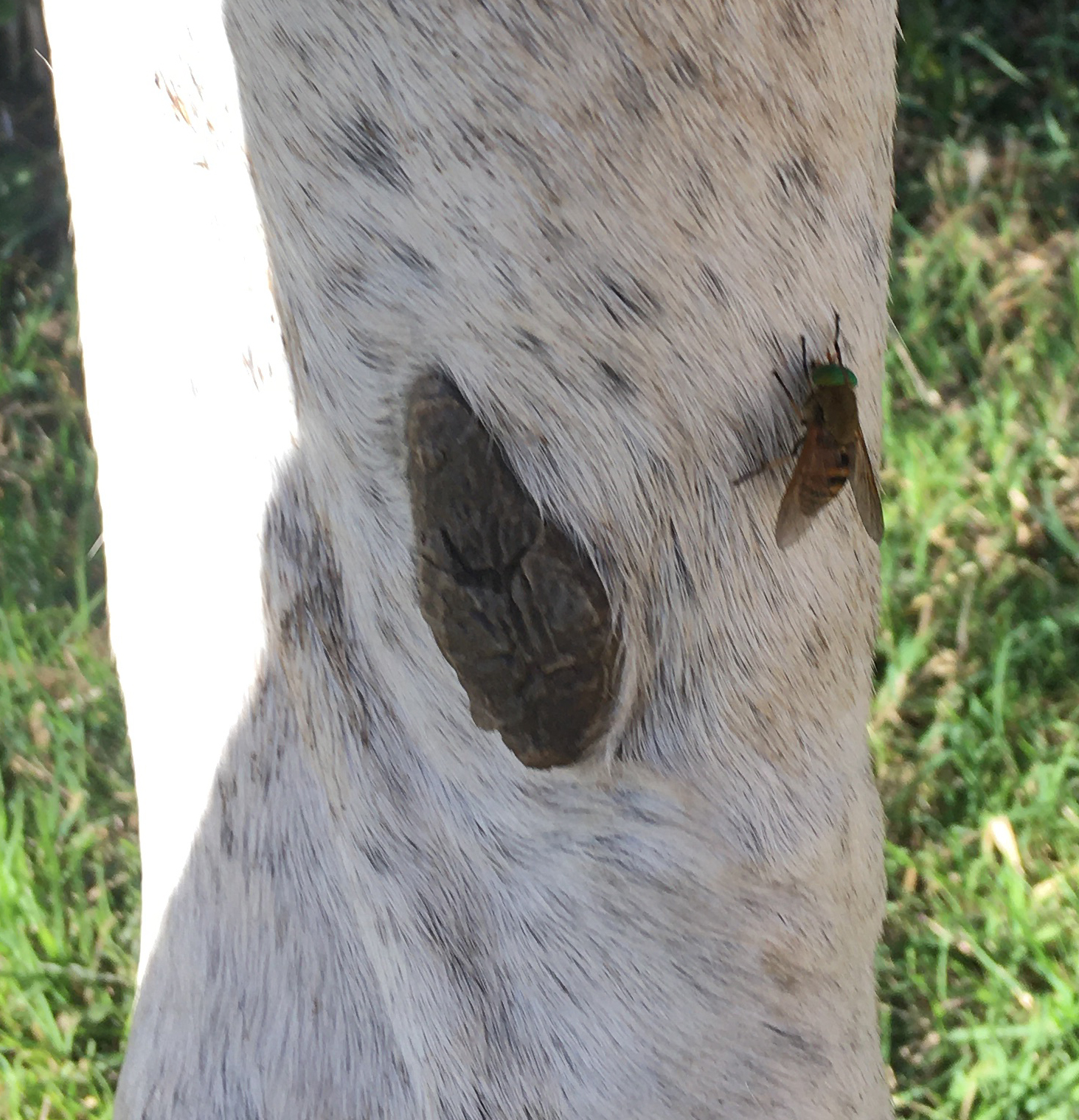

Supplement: Supplementary file 2 — Supplementary file2 (JPEG 1361 KB) [file 436_2023_8004_MOESM2_ESM.jpeg]

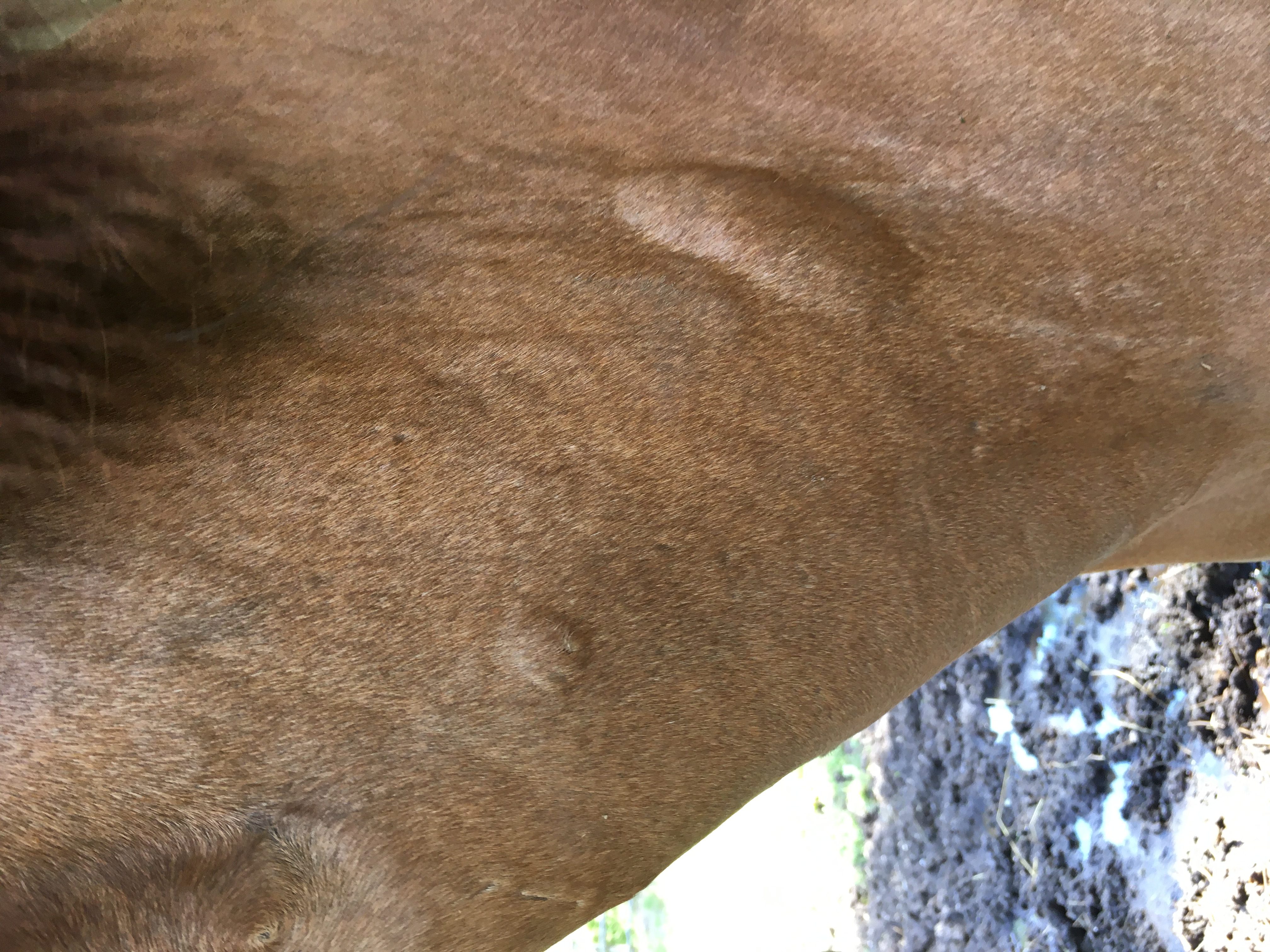

Supplement: Supplementary file 3 — Supplementary file3 (JPEG 3197 KB) [file 436_2023_8004_MOESM3_ESM.jpeg]

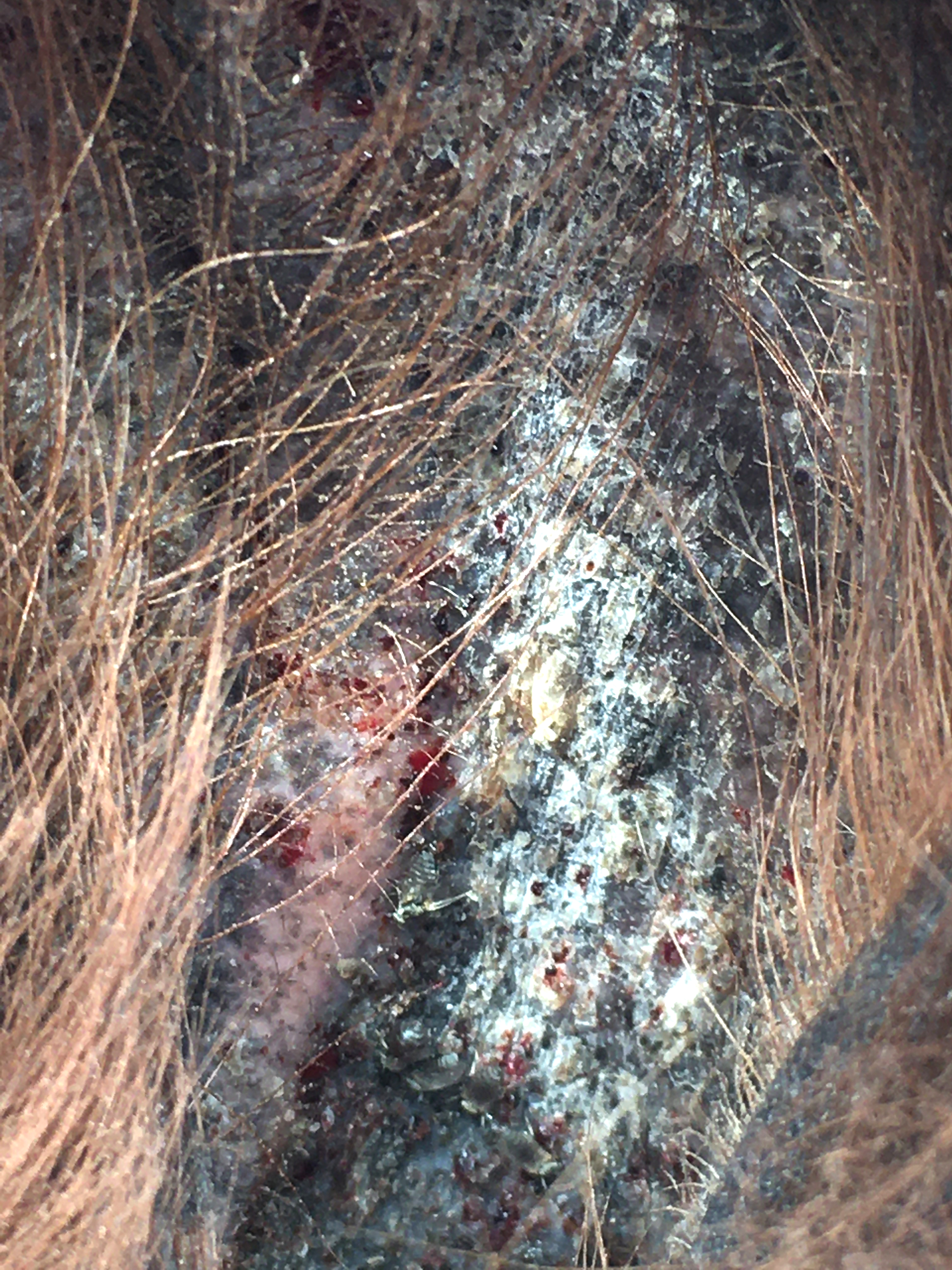

Supplement: Supplementary file 4 — Supplementary file4 (JPEG 5899 KB) [file 436_2023_8004_MOESM4_ESM.jpeg]

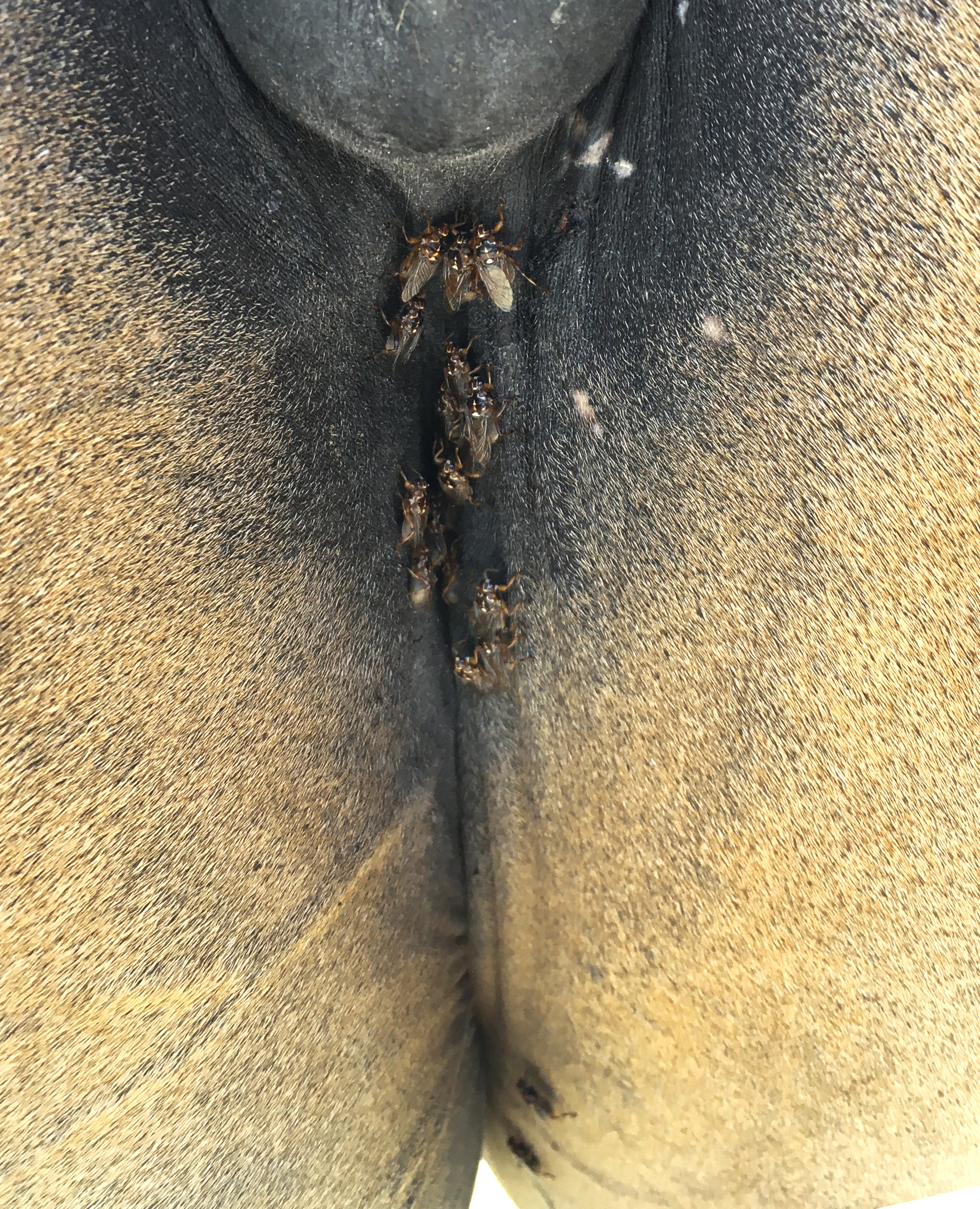

Supplement: Supplementary file 5 — Supplementary file5 (JPEG 5313 KB) [file 436_2023_8004_MOESM5_ESM.jpeg]
