# Supplementary material for: Efficacy of topical administration of prallethrin-permethrin-piperonyl butoxide (Bronco® Equine Fly Spray) for the treatment and control of flies and other nuisance insects of horses
Source: Parasitol Res. 2023 Nov 3;122(12):3139–45. doi: 10.1007/s00436-023-08004-0 (PMC10667147; doi:10.1007/s00436-023-08004-0)
Supplement: Supplementary file 6 — Supplementary file6 (DOCX 38 KB) [file 436_2023_8004_MOESM6_ESM.docx]

Table ST1A. Average values of the counts for each horse and **for control** group at Day 0 (ID 7, 8, 9). Legend: *Mu d:* *Musca domestica*. *Mu a:* *M.* *autumnalis. Hipp*: *Hippobosca equina.* *Tab*: Tabanidae*.* *Sim*: *Simulium* spp.

|  |  | ID 7 | | | | | ID 8 | | | | | ID 9 | | | | |
| --- | --- | --- | --- | --- | --- | --- | --- | --- | --- | --- | --- | --- | --- | --- | --- | --- |
| Day | **Time** | *Mu d* | *Mu a* | *Hipp* | *Tab* | *Sim* | *Mu d* | *Mu a* | *Hipp* | *Tab* | *Sim* | *Mu d* | *Mu a* | *Hipp* | *Tab* | *Sim* |
| 0 | 10:00 | 16 | 4 | 23 | 3 | 5 | 14 | 0 | 21 | 2 | 4 | 25 | 3 | 8 | 3 | 4 |
|  |  | *Treatment* | | | | | *Treatment* | | | | | *Treatment* | | | | |
|  | 1' | 11 | 4 | 23 | 2 | 5 | 14 | 0 | 21 | 1 | 4 | 25 | 3 | 8 | 1 | 5 |
|  | 10' | 15 | 6 | 23 | 1 | 5 | 9 | 0 | 21 | 1 | 5 | 20 | 4 | 8 | 0 | 5 |
|  | 20' | 9 | 5 | 23 | 1 | 7 | 11 | 1 | 18 | 2 | 4 | 10 | 8 | 8 | 2 | 5 |
|  | 30' | 16 | 10 | 20 | 3 | 8 | 15 | 1 | 18 | 3 | 6 | 16 | 8 | 8 | 2 | 4 |
|  | 1h | 11 | 12 | 15 | 2 | 7 | 14 | 3 | 16 | 2 | 5 | 11 | 12 | 7 | 3 | 8 |
|  | 2h | 9 | 11 | 16 | 3 | 18 | 7 | 4 | 19 | 6 | 5 | 8 | 14 | 12 | 3 | 16 |
|  | 3h | 16 | 15 | 19 | 2 | 11 | 12 | 3 | 19 | 3 | 3 | 14 | 15 | 11 | 4 | 11 |
|  | 4h | 21 | 15 | 15 | 1 | 9 | 16 | 6 | 15 | 2 | 5 | 17 | 9 | 10 | 0 | 8 |
|  | 5h | 16 | 9 | 14 | 1 | 11 | 21 | 3 | 13 | 1 | 5 | 20 | 6 | 9 | 1 | 10 |
|  | 6h | 13 | 6 | 21 | 3 | 11 | 13 | 3 | 21 | 5 | 2 | 14 | 7 | 13 | 4 | 11 |
|  | Total amount | 137 | 93 | 189 | 19 | 92 | 132 | 24 | 181 | 26 | 44 | 155 | 86 | 94 | 20 | 83 |
|  | Average value | 13.7 | 9.3 | 18.9 | 1.9 | 9.2 | 13.2 | 2.4 | 18.1 | 2.6 | 4.4 | 15.5 | 8.6 | 9.4 | 2.0 | 8.3 |

Table ST1B. Average values of the counts for each horse and for the **spray treatment group** at Day 0 (ID 1, 2, 3, 4, 5). Legend: *Mu d:* *Musca domestica*. *Mu a:* *M.* *autumnalis. Hipp*: *Hippobosca equina.* *Tab*: Tabanidae*.* *Sim*: *Simulium* spp.

| Day | Time | ID 1 | | | | | ID 2 | | | | | ID 3 | | | | |
| --- | --- | --- | --- | --- | --- | --- | --- | --- | --- | --- | --- | --- | --- | --- | --- | --- |
|  |  | *Mu d* | *Mu a* | *Hipp* | *Tab* | *Sim* | *Mu d* | *Mu a* | *Hipp* | *Tab* | *Sim* | *Mu d* | *Mu a* | *Hipp* | *Tab* | *Sim* |
| 0 | 10:00 | 29 | 1 | 10 | 1 | 4 | 9 | 2 | 7 | 1 | 3 | 19 | 3 | 15 | 1 | 3 |
|  |  | Treatment | | | | | Treatment | | | | | Treatment | | | | |
|  | 1' | 0 | 0 | 0 | 0 | 0 | 0 | 0 | 0 | 0 | 0 | 0 | 0 | 0 | 0 | 0 |
|  | 10' | 7 | 0 | 0 | 0 | 0 | 1 | 0 | 0 | 0 | 0 | 0 | 0 | 0 | 0 | 0 |
|  | 20' | 6 | 0 | 0 | 0 | 0 | 1 | 0 | 0 | 0 | 0 | 0 | 0 | 0 | 0 | 0 |
|  | 30' | 5 | 1 | 0 | 0 | 0 | 0 | 0 | 0 | 0 | 0 | 0 | 1 | 0 | 0 | 0 |
|  | 1h | 3 | 0 | 0 | 0 | 0 | 0 | 0 | 0 | 0 | 0 | 1 | 1 | 0 | 0 | 0 |
|  | 2h | 1 | 1 | 0 | 0 | 0 | 3 | 0 | 0 | 0 | 0 | 0 | 5 | 0 | 0 | 0 |
|  | 3h | 3 | 6 | 0 | 0 | 0 | 3 | 0 | 0 | 0 | 0 | 5 | 0 | 0 | 0 | 0 |
|  | 4h | 11 | 0 | 0 | 0 | 0 | 1 | 0 | 0 | 0 | 0 | 9 | 0 | 0 | 0 | 0 |
|  | 5h | 8 | 0 | 0 | 0 | 0 | 1 | 0 | 0 | 0 | 0 | 9 | 0 | 0 | 0 | 0 |
|  | 6h | 11 | 0 | 0 | 0 | 0 | 3 | 0 | 0 | 0 | 0 | 5 | 0 | 0 | 0 | 0 |
|  | Total amount | 55 | 8 | 0 | 0 | 0 | 13 | 0 | 0 | 0 | 0 | 29 | 7 | 0 | 0 | 0 |
|  | Average value | 5.5 | 0.8 | 0.0 | 0.0 | 0.0 | 1.3 | 0.0 | 0.0 | 0.0 | 0.0 | 2.9 | 0.7 | 0.0 | 0.0 | 0.0 |

| Day | Time | ID 4 | | | | | ID 5 | | | | |
| --- | --- | --- | --- | --- | --- | --- | --- | --- | --- | --- | --- |
|  |  | *Mu d* | *Mu a* | *Hipp* | *Tab* | *Sim* | *Mu d* | *Mu a* | *Hipp* | *Tab* | *Sim* |
| 0 | 10:00 | 10 | 4 | 35 | 4 | 2 | 19 | 1 | 9 | 3 | 6 |
|  |  | Treatment | | | | | Treatment | | | | |
|  | 1' | 0 | 0 | 0 | 0 | 0 | 0 | 0 | 0 | 0 | 0 |
|  | 10' | 0 | 0 | 0 | 0 | 0 | 0 | 0 | 0 | 0 | 0 |
|  | 20' | 0 | 0 | 0 | 0 | 0 | 0 | 0 | 0 | 0 | 0 |
|  | 30' | 0 | 0 | 0 | 0 | 0 | 0 | 0 | 0 | 0 | 0 |
|  | 1h | 0 | 0 | 0 | 0 | 0 | 0 | 0 | 0 | 0 | 0 |
|  | 2h | 0 | 0 | 0 | 0 | 0 | 0 | 0 | 0 | 0 | 0 |
|  | 3h | 5 | 0 | 0 | 0 | 0 | 8 | 0 | 0 | 0 | 0 |
|  | 4h | 5 | 0 | 0 | 0 | 0 | 5 | 0 | 0 | 0 | 0 |
|  | 5h | 5 | 0 | 0 | 0 | 0 | 7 | 0 | 0 | 0 | 0 |
|  | 6h | 6 | 0 | 0 | 0 | 0 | 6 | 0 | 0 | 0 | 0 |
|  | Total amount | 21 | 0 | 0 | 0 | 0 | 26 | 0 | 0 | 0 | 0 |
|  | Average value | 2.1 | 0 | 0 | 0 | 0 | 2.6 | 0 | 0 | 0 | 0 |

Table ST1C. Average values of the counts for each horse and **for the cloth treatment** group at Day 0 (ID 6). Legend: *Mu d:* *Musca domestica*. *Mu a:* *M.* *autumnalis. Hipp*: *Hippobosca equina.* *Tab*: Tabanidae*.* *Sim*: *Simulium* spp.

| Day | Time | ID 6 | | | | |
| --- | --- | --- | --- | --- | --- | --- |
|  |  | *Mu d* | *Mu a* | *Hipp* | *Tab* | *Sim* |
| 0 | 10:00 | 10 | 4 | 15 | 1 | 3 |
|  |  | Treatment | | | | |
|  | 1' | 2 | 0 | 3 | 0 | 0 |
|  | 10' | 2 | 0 | 0 | 0 | 0 |
|  | 20' | 2 | 0 | 0 | 0 | 0 |
|  | 30' | 0 | 0 | 0 | 0 | 0 |
|  | 1h | 0 | 0 | 0 | 0 | 0 |
|  | 2h | 0 | 0 | 0 | 0 | 0 |
|  | 3h | 0 | 0 | 0 | 0 | 0 |
|  | 4h | 0 | 0 | 0 | 0 | 0 |
|  | 5h | 0 | 0 | 0 | 0 | 0 |
|  | 6h | 0 | 0 | 0 | 0 | 0 |
|  | Total amount | 6 | 0 | 3 | 0 | 0 |
|  | Average value | 0.6 | 0 | 0.3 | 0 | 0 |
